# Effects of exogenous spermidine on the morphology and physiology of two ecological grasses under flooding stress

**DOI:** 10.3389/fpls.2025.1683739

**Published:** 2025-12-09

**Authors:** Rui Sun, Hui Hu, Die Hu, Yujie Yang, Zihan Cheng, Xiaohu Chen, Yongjun Fei

**Affiliations:** 1 Hubei Key Laboratory of Spices & Horticultural Plant Germplasm Innovation & Utilization, Yangtze University, Jingzhou, China; 2 Germplasm Resources Evaluation and Innovation Center of Phoebe Nees and Machilus Nees, College of Horticulture and Gardening, Yangtze University, Jingzhou, China

**Keywords:** flooding stress, waterlogging tolerance, exogenous spermidine, *Elytrigia elongata*, *Cynodon dactylon* × C. *transvaalensis ‘Tifdwarf’*

## Abstract

This study used *Elytrigia elongata* and *Cynodon dactylon × C. transvaalensis ‘Tifdwarf’* as experimental materials. Flooding stress was simulated by raising the water level 2 cm above the soil surface, while the control (CK) involved conventional water management. Three treatments were applied: flooding stress alone (FS), and flooding combined with 1 mmol/L spermidine (FP). Each treatment lasted for 5, 10 and 20 days. The aim was to investigate the impact of 1 mmol/L spermidine (Spd) on the growth and physiological responses of seedlings of both species subjected to different durations of flooding stress. The results showed that applying exogenous Spd under flooding conditions significantly increased the number of epidermal cells and biomass accumulation. Spd increased photosynthetic capacity by raising chlorophyll content and gas exchange parameters while reducing antioxidant enzyme activity. This elevated free-state Spd levels and enabled the removal of accumulated reactive oxygen species (ROS). Additionally, Spd promoted the accumulation of osmoregulatory substances, alleviating oxidative damage to membrane lipids and maintaining cellular osmotic potential. Consequently, Spd mitigated the adverse effects of flooding stress on plant growth. Correlation and principal component analyses of physiological and biochemical indicators confirmed that exogenous Spd improved flooding tolerance in both species. Notably, *Elytrigia elongata* exhibited greater tolerance to flooding, and the mitigating effects of Spd were more pronounced in this species under the same inundation conditions. Overall, this study provides a theoretical foundation for mitigating flooding induced damage in ecological grasses and offers insights into cultivating flooding tolerant grass species.

## 1 Introduction

In recent years, global warming has led to increased frequency of flooding during the rainy season, subjecting plants to prolonged waterlogging stress and severely threatening ecological stability (Qiaoyu et al., 2023). Flooding induces oxygen deficiency in plant roots, disrupting normal respiration and subsequently impairing photosynthesis and other essential physiological processes (Wei, 2009). Under hypoxic conditions, the absorption of mineral nutrients from the soil is markedly reduced, causing a decline in cellular physiological functions and energy metabolism. This often results in root rot and lignification, ultimately inhibiting plant growth and development (Wei, 2009). Under waterlogging stress, the triggered oxidative burst inflicts membrane damage and lipid peroxidation, thereby activating the plant’s antioxidant enzyme system as a defense mechanism to scavenge reactive oxygen species (ROS) (Yang et al., 2015); Additionally, they adapt by forming adventitious roots and aerenchyma to facilitate internal aeration (Jackson and Colmer, 2005), and by accumulating osmoregulatory substances to reduce cellular water uptake and maintain osmotic balance (Pang et al., 2018).

Spermidine (Spd) is a naturally occurring polyamine widely present in both animals and plants (Liu et al., 2011; Cheng et al., 2010). It is a critical component of polyamines in plant tissues and plays a direct role in physiological processes such as growth, cell division, senescence, and stress responses (Mattoo et al., 2010). Among various abiotic stresses, exogenous application of Spd is indispensable for enhancing plant growth and improving resistance to environmental challenges, functioning as an important bioactive compound (Zhang et al., 2015). Specifically, Spd plays an important role in mitigating flooding stress. For example, external application of Spd enhances waterlogging tolerance in *Axonopus compressus*, reducing damage under flooding stress (Yang et al., 2025). In Fujian mountain cherry trees, Spd treatment increases peroxidase (POD) activity, decreases malondialdehyde (MDA), soluble sugar, and soluble protein content, thereby alleviating membrane damage caused by flooding stress (Lu et al., 2022). Similarly, Spd application improves photosynthetic capacity and waterlogging tolerance in maize leaves, resulting in increased dry matter accumulation (Cui et al., 2022). Additionally, Spd enhances antioxidant capacity and osmotic regulation in maize seedlings under flooding stress, and effectively improves physiological functions in maize roots and leaves, ultimately reducing yield losses (You et al., 2016; Seng et al., 2012a).

*Elytrigia elongata*, a herbaceous species in the Poaceae family and *Elytrigia* genus, possesses a well-developed root system, high biomass, and favorable nutritional quality (Li et al., 2022). It demonstrates strong adaptability to diverse growth conditions, exhibiting notable tolerance to saline alkaline soils, flooding, and drought, making it highly valuable for ecological environmental protection (Li et al., 2023), However, research on its physiological responses to flooding stress remains limited. *Cynodon dactylon × C. transvaalensis ‘Tifdwarf’* (Xuan et al., 2005), commonly known as putting green grass due to its widespread use in golf course greens, is a hybrid of common bermudagrass and African bermudagrass (Zhou and Liu, 2016). As an evergreen perennial grass of the Poaceae family and Cynodon genus, this species exhibits vigorous growth and a suite of exceptional stress tolerances, including resistance to salt-alkali, drought, barren soil, lodging, close mowing, and wear, These combined characteristics account for its widespread application in sports fields and landscape greening (Reasor et al., 2016). This study employed *Elytrigia elongata* and *Cynodon dactylon × C. transvaalensis ‘Tifdwarf’* as experimental materials to assess the effects of exogenous Spd on seedling growth and physiological responses under flooding stress. The results demonstrated that foliar application of Spd significantly enhanced waterlogging tolerance in both species, providing a theoretical foundation for cultivating flood resistant grasses and promoting ecological restoration.

## 2 Materials and methods

### 2.1 Test materials

The experiment was conducted in 2023 within a greenhouse located at the West Campus of Yangtze University in Jingzhou District, Jingzhou City, Hubei Province (30°21′N, 112°08′E). Throughout the study, greenhouse conditions were maintained with temperatures ranging 18 to 25°C, relative humidity was 80%, and natural light provided from 8:00 to 18:00. The test materials comprised seeds of the *Elytrigia elongata* obtained from Crowvo (Beijing) Ecological Technology Co., Ltd., seeds of *Cynodon dactylon × C. transvaalensis ‘Tifdwarf’* sourced from Shuyang Mipei Seed Industry Co., Ltd., and spermidine purchased from Sigma.

Three treatments were established for each grass species: normal moisture treatment (CK), flooding treatment (FS), and flooding combined with spermidine application (FP). Each treatment included nine replicates, with 35 plants per replicate, totaling 27 experimental units per species. In June 2023, a preliminary experiment was conducted to evaluate the efficacy of exogenous spermidine at concentrations of 0.25, 0.5, and 1 mmol/L in mitigating flooding stress on the two grass species. After 20 days of treatment, leaf yellowing and senescence were observed. Subsequently, chlorophyll a, chlorophyll b, and plant height were measured, followed by membership function analysis. The results ranked the treatments as follows: 1 mmol/L > 0.25 mmol/L > 0.5 mmol/L, supporting the selection of 1 mmol/L spermidine for a 20-day treatment period. After one month, seeds were disinfected and evenly sown at 50–80 seeds per plastic pot filled with substrate. When seedlings reached the three leaf stage, the main experiment commenced to assess the effects of exogenous Spd on the response of the two ecological grasses to waterlogging stress.

### 2.2 Experimental design

Plump and uniformly sized seeds were selected and soaked in a 500-fold dilution of Mefenoxam for 6 hours to ensure sterilization and disinfection. After treatment, the seeds were thoroughly rinsed with water and air dried for later use. Mefenoxam was sourced from Hebei Zhongnong Lvbao Crop Technology Co., Ltd. The planting substrate, composed of pond soil and river sand in a 1:1 ratio, was sterilized using a high-pressure steam autoclave (0.11 MPa, 121°C) for 2 hours to eliminate potential pathogens, weed seeds, and insects prior to sowing.

For water management, the control group (CK) was irrigated daily using the weighing method to maintain soil moisture at 60%–75%. Flooding treatments were applied using the pot immersion method, in which pots were placed in plastic basins (73 cm × 36.5 cm × 21 cm) with water maintained at 2 cm above the soil surface. During the treatment period, the CK and FS groups were watered daily between 17:00 and 18:00. The FP group received foliar applications of 1 mmol·L^−1^ exogenous spermidine, sprayed until the leaf surfaces were fully wetted and dripping. Sampling was conducted on days 5, 10, and 20 of the flooding treatment and designated as CK-5, FS-5, FP-5; CK-10, FS-10, FP-10; and CK-20, FS-20, FP-20, respectively. At each time point, three replicates were randomly selected for measurements of biomass, photosynthetic parameters, and root morphology. Additional plant samples were stored at –20°C for further analysis of physiological and biochemical indicators.

### 2.3 Leaf epidermal sections, biomass, and root morphology

Randomly select 10 leaves each from both ecological grass species subjected to 5, 10, and 20 days of flooding treatment were randomly selected. A 10 mm segment from the middle portion of the leaf was excised and fixed in FAA fixative solution (glacial acetic acid: formaldehyde: ethanol: water = 1:1:12:6) for fixation for subsequent analysis. Fixed samples were immersed in a dissociation solution composed of 30% hydrogen peroxide and 30% acetic acid (1:1, v/v) for 6 hours, then rinsed thoroughly. The upper epidermis and mesophyll were gently scraped off using a razor blade. The remaining lower epidermis was flattened with forceps, stained with 1% safranin solution, and rinsed to remove excess dye. Then observe and photograph under a microscope, and use ImageJ to count the number of epidermal cells.

After the completion of each flooding period, 10 plants per treatment group were randomly selected. Roots were gently washed to remove soil, and shoots were rinsed to remove surface dust. The above ground and belowground parts were dried with absorbent paper and weighed separately using a precision electronic balance (accuracy: 0.001 g). Root morphological parameters were assessed using an EPSON Scan v3.771 scanner (Japan) at 400 dpi resolution. The resulting root images were analyzed using WinRHIZO Pro 2007a software to determine total root length (cm).

### 2.4 Photosynthetic pigment content and photosynthetic gas exchange parameters

On the 5th, 10th and 20th days of flooding treatment, healthy and fully expanded leaves with similar light exposure were selected to measure gas exchange parameters. Net photosynthetic rate (Pn), stomatal conductance (Gs), intercellular CO_2_ concentration (Ci), and transpiration rate (Tr) were measured between 13:00 and 14:00—when light intensity was at its peak—using a LI-6400 portable photosynthesis system (LI-COR Bio-sciences, USA). For each treatment, 10 plants were measured, with three readings taken per leaf (Wu, 2018); The contents of chlorophyll a, chlorophyll b, and carotenoids were determined using the ethanol extraction method. For each treatment, 0.2 g of fresh leaf tissue was sampled, with three biological replicates performed per treatment (Wu, 2018).

### 2.5 Measurement of physiological indicators

Take 0.3 grams of fresh leaf tissue from each of the two ecological grass seedlings for physiological parameter measurements. Each treatment should be performed with three biological replicates. Physiological indexes were measured by spectrophotometric method (Wu, 2018) using leaves of two ecograss seedlings as samples to determine Superoxide dismutase (SOD) activity、Peroxidase (POD) activity、Catalase (CAT) activity、Ascorbate peroxidase (APX) activity、 Malondialdehyde (MDA) content,、free proline (Pro) content; titanium sulfate colorimetric method to determine the content of hydrogen peroxide (H_2_O_2_) (Liu et al., 2000); Superoxide anion radical (O_2_^−^) content was determined by the method of Wu Qiangsheng et al (Wu, 2018); soluble protein was determined by the colorimetric method of Caulmers Brilliant Blue G-250 (Wu, 2018).

### 2.6 Data analysis

Experimental data were organized and processed using Microsoft Office Excel 2019. Statistical analyses, including one way ANOVA and Duncan’s multiple comparison tests, were conducted in IBM SPSS Statistics 20 to evaluate significant differences among treatments, with significance defined at p < 0.05. Graphical representations were generated using Origin2022. To comprehensively assess the morphological and physiological responses of the two ecological grasses to exogenous spermidine (Spd) under flooding stress, data were further analyzed using flood tolerance coefficients, principal component analysis (PCA), and membership function methods.

## 3 Results

### 3.1 Effects of exogenous Spd on epidermal cells numbers in two ecological grasses under flooding stress

**Figure 1:**
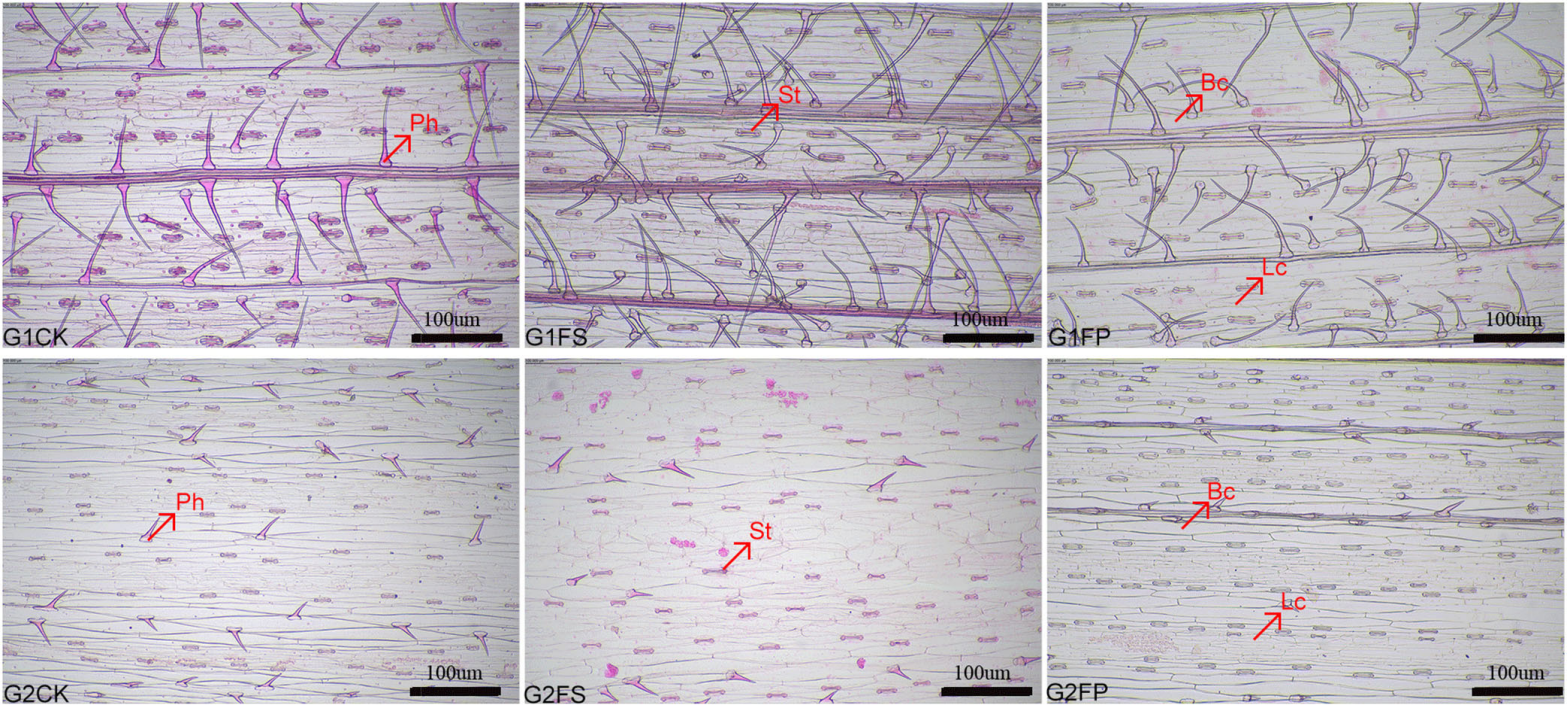
Preparation of the epidermis of *Elytrigia elongata* and *Cynodon dactylon × C. transvaalensis* ‘Tifdwarf’. scale=100 μm; G1. *Elytrigia elongata*, G2. *Cynodon dactylon × C. transvaalensis* ‘Tifdwarf’; CK—control, FS—waterlogging, FP—waterlogging+Spd; Ph: Prickle-hair; St: Stoma; Lc: long cell; Bc: Buffiform cell.

Spd influenced the number of stomata, prickle-hair, long cells, and bulliform cells in both ecological grasses (**Table 1**). Flooding stress significantly reduced the counts of these epidermal cell types in both species. However, application of exogenous Spd markedly increased (p < 0.05) the numbers of stomata and prickle hairs in *E. elongata*. In *‘Tifdwarf’*, Spd treatment led to significant increases in the numbers of stomata, prickle hairs, and long cells (**Figure 1**).

**Table 1:** Numerical characteristics of lower epidermal cells in two Ecological grasses.

Treatment	Number of stoma/pcs	Number of prickle-hair/pcs	Number of long cells/pcs	Number of buffiform cell/pcs
G1CK	66.00±3.46a	84.33±11.55a	65.67±24.85a	109.33±19.63a
G1FS	50.67±2.08b	55.67±3.06b	45.67±8.02a	77.00±18.52a
G1FP	59.33±6.11a	89.00±11.36a	62.00±19.97a	134.67±53.43a
G2CK	75.33±6.35b	31.00±5.20b	84.00±12.29a	131.33±11.02a
G2FS	60.67±3.21c	15.33±2.52c	41.00±14.93b	109.67±8.14a
G2FP	95.67±4.93a	39.33±1.53a	78.67±5.13a	118.67±21.2a

G1. *Elytrigia elongata*, G2. *Cynodon dactylon × C. transvaalensis* ‘Tifdwarf’, CK—control, FS—waterlogging, FP—waterlogging+Spd; a, b, c, represent p<0.05.

### 3.2 Effect of exogenous Spd on biomass of two ecological grasses under flooding stress

**Figure 2:**
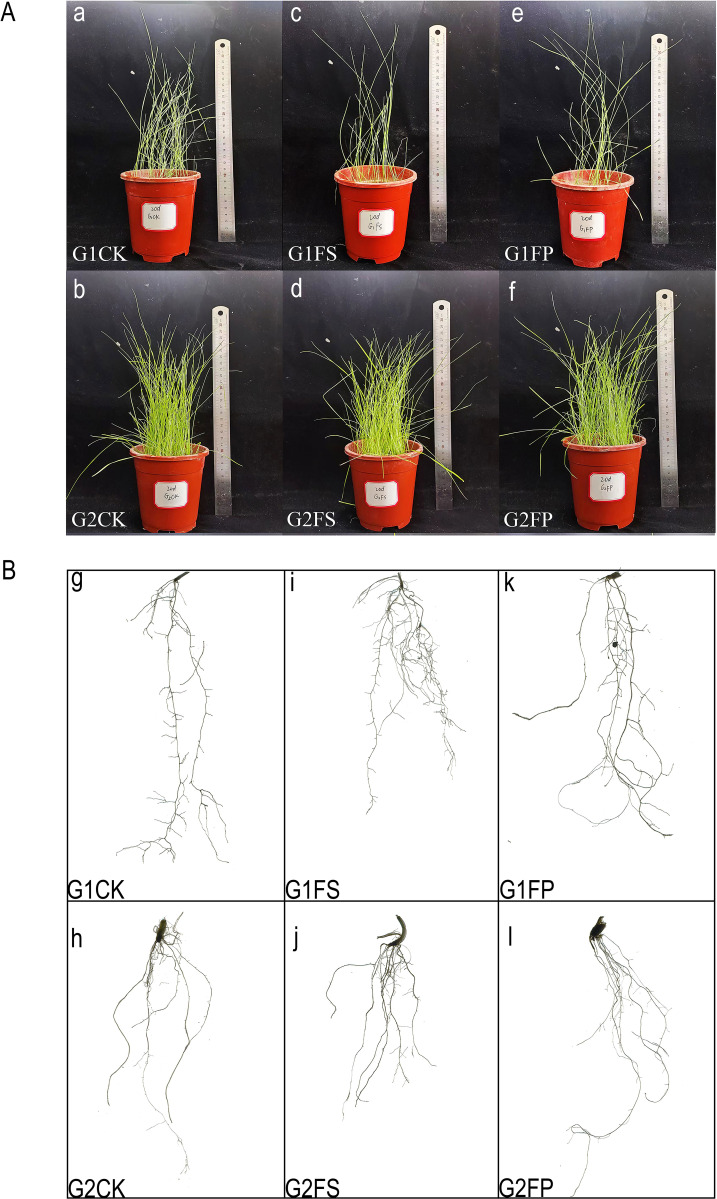
Effect of exogenous Spd on biomass of two ecological grasses under flooding stress. **(A)** a~f: Growth of two Ecological grasses under different treatments after 20 days; **(B)** g~i: Root Scanning of Two Ecological Grasses After 20 Days Under Different Treatments; G1. *Elytrigia elongata*, G2. *Cynodon dactylon × C. transvaalensis* ‘Tifdwarf’, CK—control, FS—waterlogging, FP—waterlogging+Spd. .

After 20 days of flooding stress, the above ground parts of *E. elongata* were severely affected, resulting in a reduction in plant numbers. However, exogenous application of Spd significantly increased both plant count and height compared to the FS treatment alone. In contrast, *‘Tifdwarf’* exhibited no visible adverse effects from flooding. Nevertheless, the number of plants increased following exogenous Spd application (**Figure 2**).

**Figure 3:**
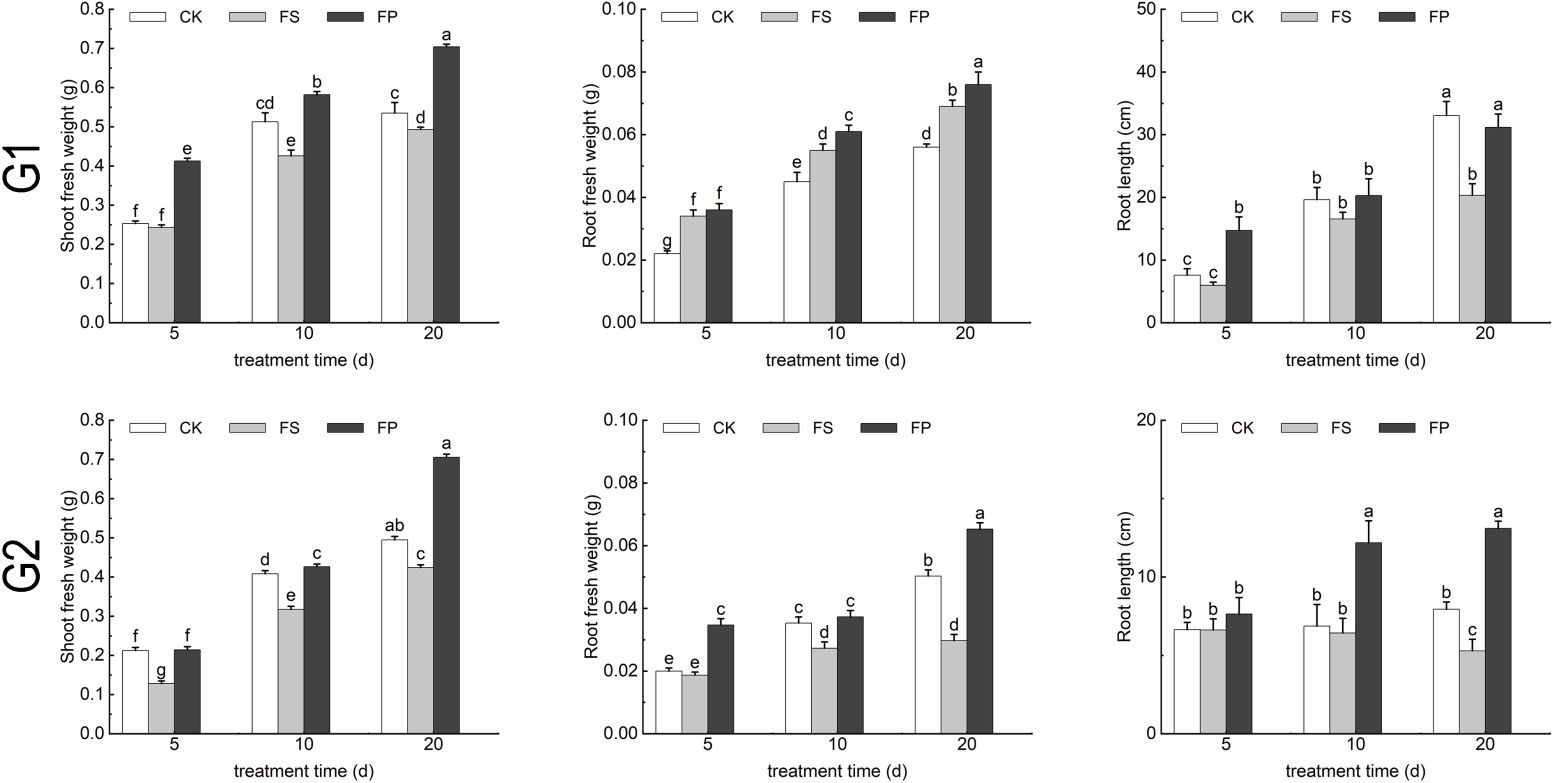
Biomass of two Ecological grasses under different treatments. G1. *Elytrigia elongata*, G2. *Cynodon dactylon × C. transvaalensis* ‘Tifdwarf’, CK—control, FS—waterlogging, FP—waterlogging+Spd; different letters above the bars indicate significant (p < 0.05) differences.

After 20 days of treatment, the application of exogenous Spd under flooding conditions (FP) promoted root length in both ecological grasses compared to flooding stress(FS) (**Figure 2**). Measurement of growth indicators under different treatments showed that flooding stress increased the root fresh weight of *E. elongata* but decreased that of ‘Tifdwarf’. In *E. elongata*, shoot fresh weight and root length under flooding stress (FS) were reduced compared to the control (CK) at 5, 10, and 20 days, with the greatest reduction in shoot fresh weight (16.96%) at 10 days and the largest decline in root length (38.52%) at 20 days. Exogenous spermidine (Spd) application increased shoot fresh weight and root length by 36.62% and 53.40%, respectively, compared to FS. Root fresh weight also increased with Spd treatment, showing the most significant rise (54.55%) at 5 days and a 5.88% increase at 20 days. For ‘Tifdwarf’, shoot fresh weight, root fresh weight, and root length all decreased under FS at all time points compared to CK, but these parameters were consistently improved by Spd application under flooding conditions (**Figure 3**).

### 3.3 Effects of exogenous spermidine on the chlorophyll content and photosynthetic parameters of two ecological grasses under flooding stress

**Figure 4:**
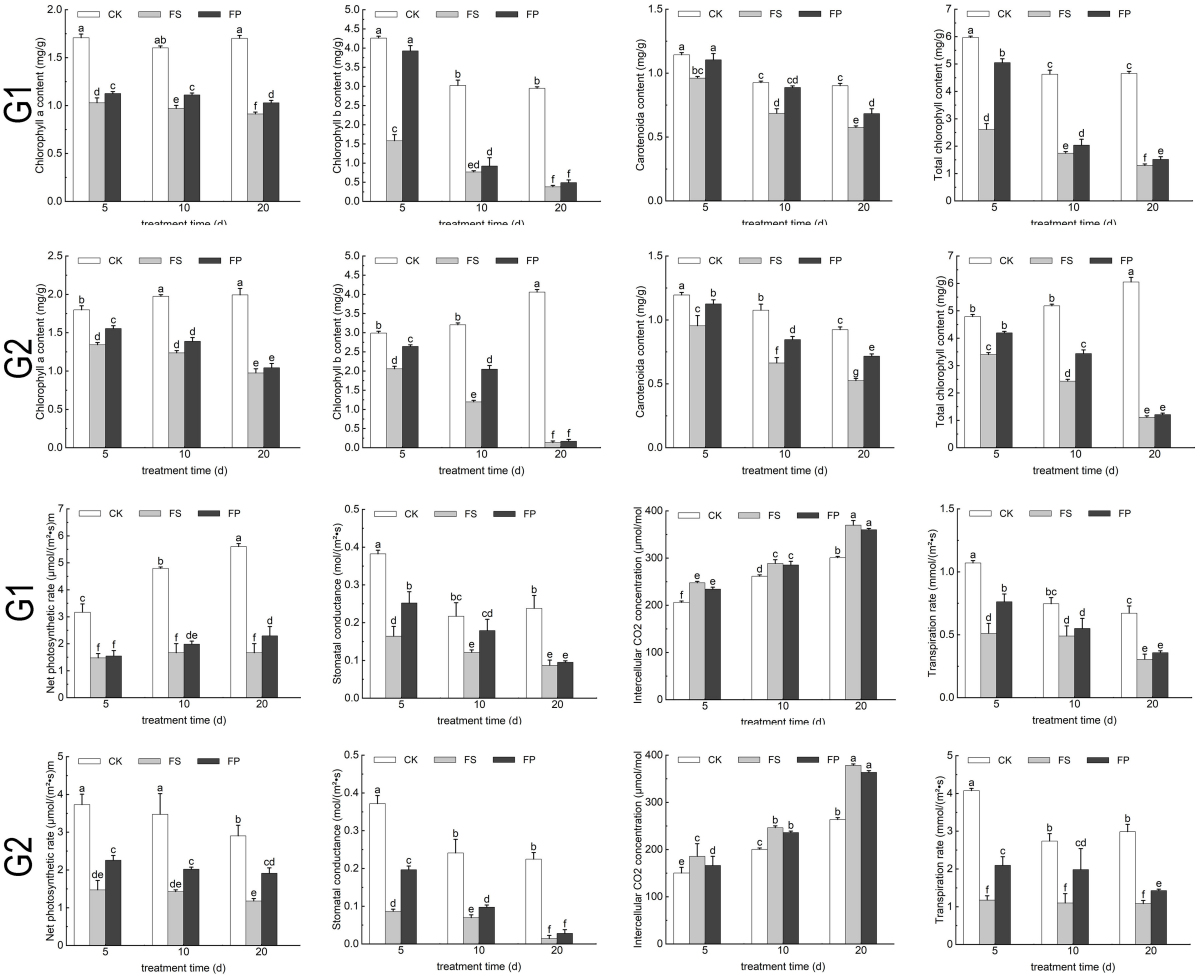
The chlorophyll content and photosynthetic parameters of two Ecological grasses under different treatments. G1. *Elytrigia elongata*, G2. *Cynodon dactylon × C. transvaalensis* ‘Tifdwarf’, CK—control, FS—waterlogging, FP—waterlogging+Spd; different letters above the bars indicate significant (p < 0.05) differences.

The chlorophyll content of the two ecological grasses under different treatments was determined. As shown in **Figure 4**, Overall, chlorophyll content followed the trend CK > FP > FS across all treatment durations. The contents of chlorophyll a, chlorophyll b, carotenoids and total chlorophyll in both of the two ecological grasses leaves showed a general decline under FS compared to the control, reaching their lowest levels after 20 days of treatment. However, the FP significantly increased the content of photosynthetic pigments relative to FS. Specifically, in *E. elongata*, chlorophyll a content under FP treatment increased by 9.53%, 14.54%, and 12.72% at 5, 10, and 20 days, respectively, compared to FS. In *‘Tifdwarf’*, the corresponding increases were 15.77%, 12.27%, and 6.90%. Similar trends were observed for chlorophyll b, carotenoids, and total chlorophyll content.

As illustrated in **Figure 4**, leaf net photosynthetic rate (Pn), stomatal conductance (Gs), and transpiration rate (Tr) in both species exhibited the trend CK > FP > FS across all treatment durations, while intercellular CO_2_ concentration (Ci) followed the opposite pattern (CK < FP < FS). Under FS, Pn, Gs, and Tr declined progressively with increasing flooding duration, reaching minimum values at 20 days, whereas Ci increased. The foliar application of Spd under flooding stress mitigated these declines in gas exchange parameters. After 5 days of treatment, *E. elongata* under FP showed increases of 4.75%, 53.66%, and 78.47% in Pn, Gs, and Tr, respectively, compared to FS (P < 0.05). *‘Tifdwarf’* exhibited even greater improvements, with increases of 53.40%, 127.55%, and 49.25%, respectively (P < 0.05). After 10 days, *E. elongata* showed increases of 19.23%, 47.93%, and 80.44%, while *‘Tifdwarf’* showed increases of 41.92%, 40.20%, and 12.24% in Pn, Gs, and Tr, respectively (P < 0.05). After 20 days, *E. elongata* exhibited increases of 37.80%, 9.20%, and 31.94%, while *‘Tifdwarf’* showed increases of 62.53%, 95.80%, and 17.30%, respectively. Although Ci values in both species under FP were lower than those under FS across all treatment durations, the differences were not statistically significant.

### 3.4 Effects of exogenous spermidine on the antioxidant systems of two ecological grasses under flooding stress

**Figure 5:**
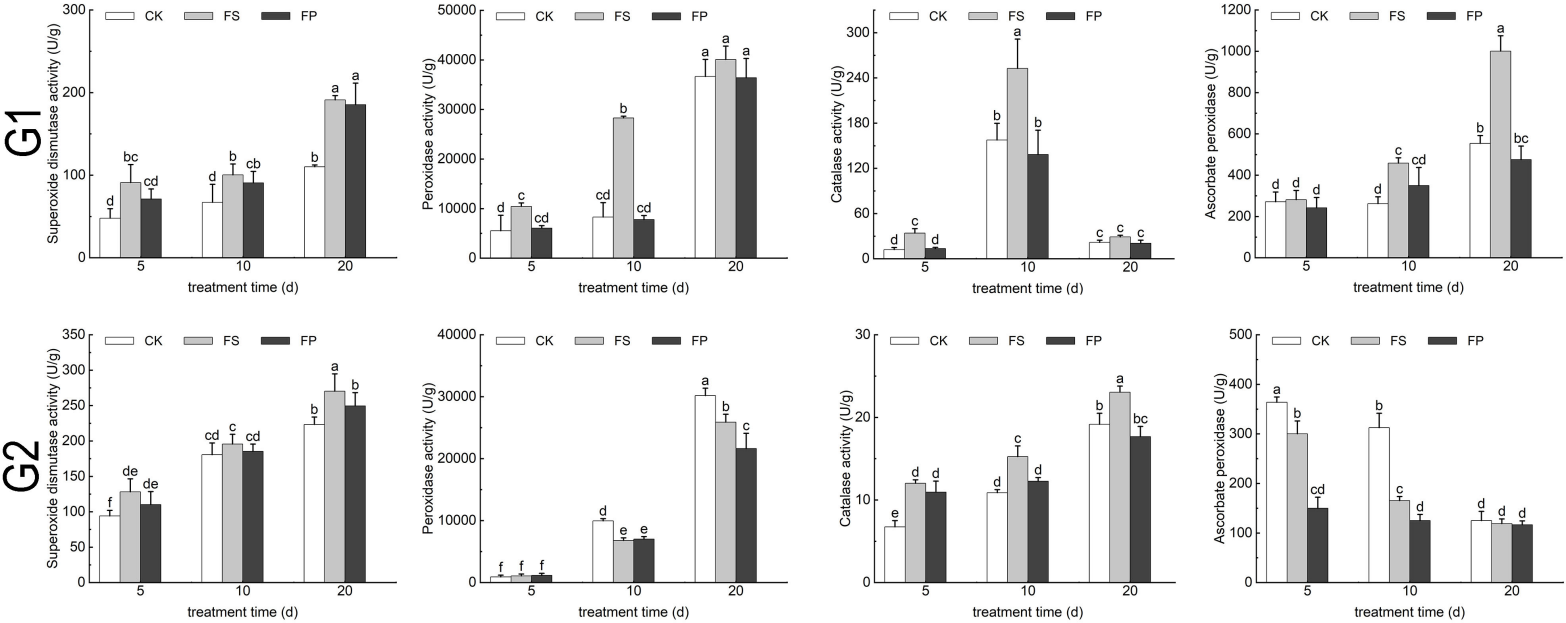
SOD、POD、CAT、APX activity of two Ecological grasses under different treatments. G1. *Elytrigia elongata*, G2. *Cynodon dactylon × C. transvaalensis* ‘Tifdwarf’, CK—control, FS—waterlogging, FP—waterlogging+Spd; different letters above the bars indicate significant (p < 0.05) differences.

Under FS treatment, the activities of SOD and CAT in both *E. elongata* and *‘Tifdwarf’* exhibited a consistent trend, increasing at days 5, 10, and 20 compared to the CK. However, following exogenous Spd application, both enzyme activities decreased relative to FS (**Figure 5**).

In *E. elongata*, the activities of peroxidase (POD) and ascorbate peroxidase (APX) increased under FS compared to CK on days 5, 10, and 20, except for a negligible change in APX activity on day 5. After Spd treatment, both POD and APX activities declined compared to FS. In *‘Tifdwarf’*, POD and APX activities generally decreased under FS relative to CK at all time points, with the exception of POD activity on day 5, which remained essentially unchanged. Following Spd application, POD activity increased by 9.08% and 3.10% compared to FS on days 5 and 10, respectively, but decreased by 16.45% on day 20. APX activity showed a continuous decline after Spd application, decreasing by 50.00%, 24.37%, and 1.75% on days 5, 10, and 20, respectively (**Figure 5**).

### 3.5 Effects of exogenous spermidine on the content of malondialdehyde and reactive oxygen species in two ecological grasses under flooding stress

**Figure 6:**
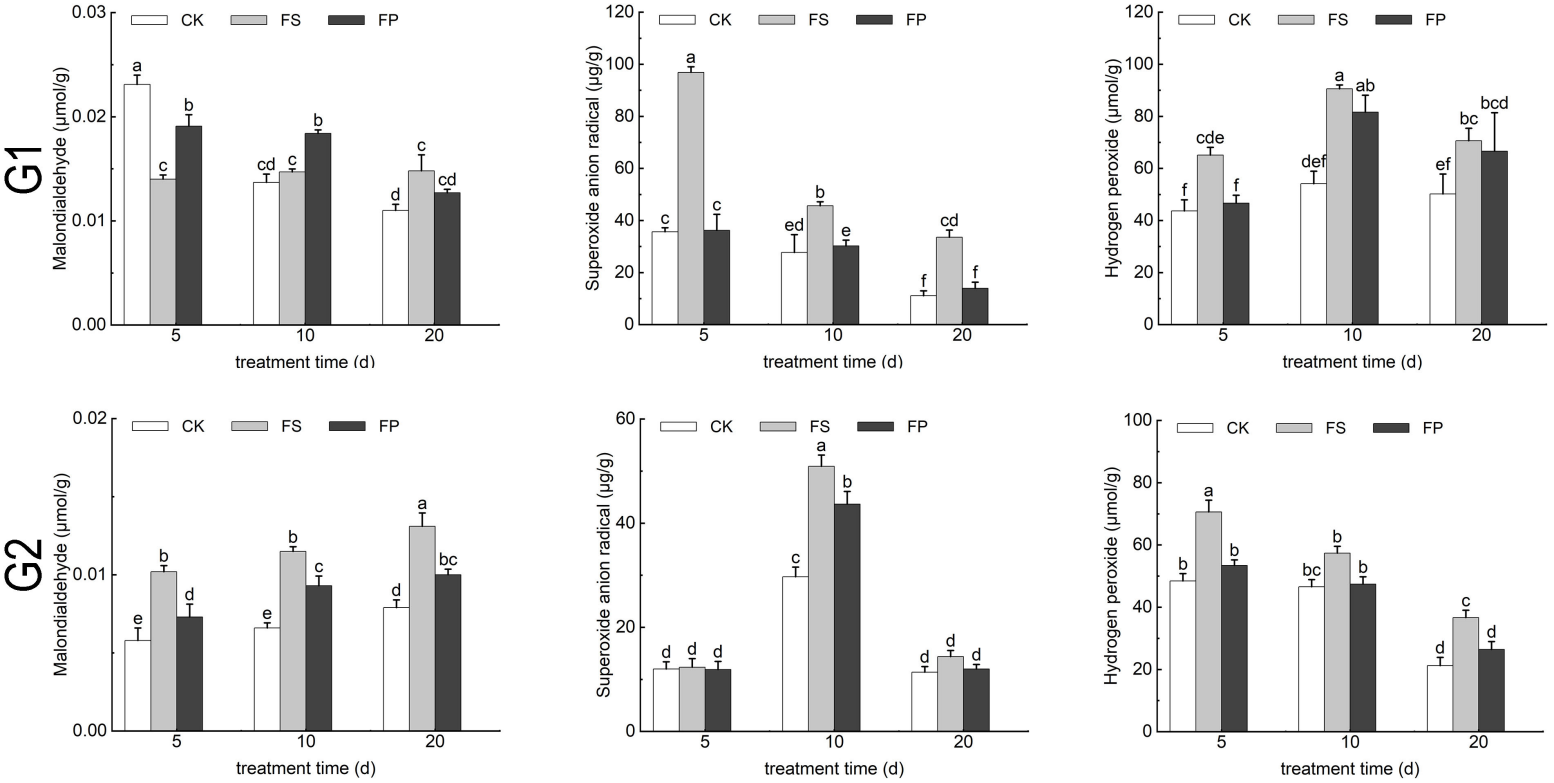
MDA content and Reactive oxygen species content of two Ecological grasses under different treatment. G1. *Elytrigia elongata*, G2. *Cynodon dactylon × C. transvaalensis* ‘Tifdwarf’, CK—control, FS—waterlogging, FP—waterlogging+Spd; different letters above the bars indicate significant (p < 0.05) differences.

Under FS treatment, the malondialdehyde (MDA) content in *E. elongata* initially decreased by 39.39% at day 5 compared to the CK, then increased by 7.30% and 34.55% at days 10 and 20, respectively. Following exogenous Spd application, MDA content exhibited a transient increase of 36.43% and 25.17% at days 5 and 10 relative to FS, followed by a significant decrease of 14.19% at day 20 (p < 0.05). In contrast, *‘Tifdwarf’* displayed an overall increase in MDA content under FS compared to CK, which was reversed to a decreasing trend after Spd treatment (**Figure 6**).

Hydrogen peroxide (H_2_O_2_) and superoxide anion radical levels in both species increased under FS relative to CK at days 5, 10, and 20. These oxidative stress markers decreased following exogenous Spd application compared to FS, roughly FS>FP>CK (**Figure 6**).

### 3.6 Effects of exogenous spermidine on the contents of free proline and soluble proteins in two ecologically grasses under flooding stress

**Figure 7:**
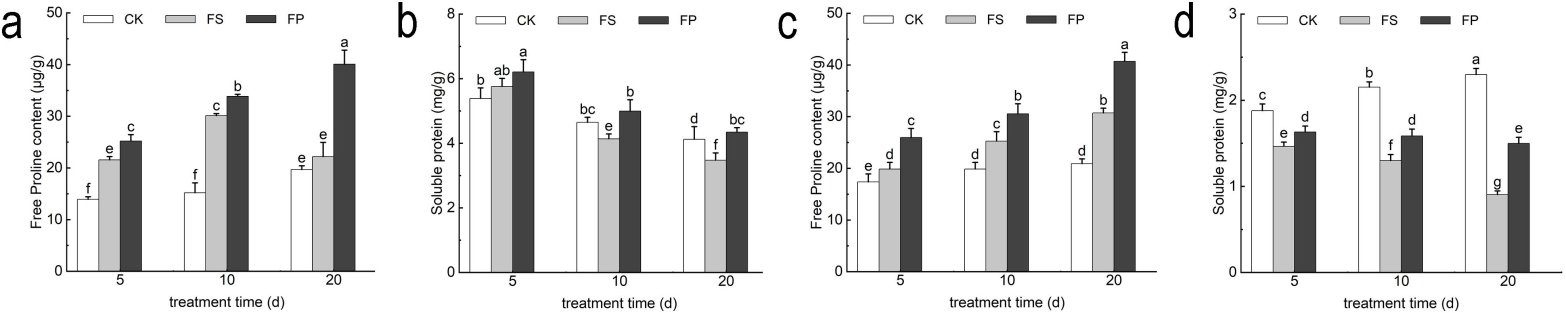
Free Proline and Soluble protein content of two Ecological grasses under different treatments. **(a)***Elytrigia elongata* free proline content; **(b)***Cynodon dactylon × C. transvaalensis* ‘Tifdwarf’ free proline content; **(c)***Elytrigia elongata* soluble protein content; **(d)***Cynodon dactylon × C. transvaalensis* ‘Tifdwarf’ soluble protein content; CK—control, FS—waterlogging, FP—waterlogging+Spd; different letters above the bars indicate significant (p < 0.05) differences.

Under flooding stress (FS), the proline (Pro) content of both *E. elongata* and *‘Tifdwarf’* showed a consistent increasing trend compared to the CK at 5, 10, and 20 days. Furthermore, the application of exogenous Spd led to a further increase in Pro content, following the pattern FP > FS > CK across all time points. For *E. elongata*, the soluble protein content under FS increased slightly by 6.81% at 5 days, but declined by 10.95% and 15.68% at 10 and 20 days, respectively, compared to CK. However, foliar application of Spd (FP) resulted in an increase in soluble protein content at all three time points compared to FS, with respective increases of 7.87%, 20.78%, and 24.93%. In contrast, *‘Tifdwarf’* exhibited a substantial decrease in soluble protein content under FS treatment, with reductions of 22.01%, 39.66%, and 60.63% at 5, 10, and 20 days, respectively, relative to CK. Following Spd application, the soluble protein content increased significantly compared to FS, by 11.37%, 22.09%, and 65.58% at the corresponding time points (**Figure 7**).

### 3.7 Principal component analysis and affiliation function analysis of flood tolerance coefficients of two ecological grasses

In this study, the principal component analysis was performed on the flooding tolerance coefficients of the two ecological grasses, with results summarized in **Table 2**. The first five principal components, explaining a cumulative variance of 91.143%, were selected for the comprehensive evaluation of flooding tolerance. Principal component 1 accounted for 36.771% of the variance and was primarily associated with chlorophyll content, as indicated by high eigenvector loadings for Carotenoid content Total chlorophyll content, shoot fresh weight and Chlorophyll b content. Principal component 2 contributed 25.431% of the variance and was mainly related to antioxidant enzyme activities, reflected by large loadings for Superoxide dismutase activity, Soluble protein content, Peroxidase activity and Ascorbate peroxidase activity. Principal component 3 explained 14.885% of the variance and was chiefly associated with osmoregulatory substances, such as shoot fresh weight and proline content (**Table 2**).

**Table 2:** Eigenvectors and contributions to principal component analysis of flooding tolerance coefficients of two ecological grasses with various indicators.

Index	Principal component				
	1	2	3	4	5
Shoot fresh weight	0.069	0.005	-0.273	-0.107	-0.012
Root fresh weight	0.111	0.069	0.012	-0.164	-0.052
Root length	0.100	-0.063	-0.113	-0.105	0.156
Chlorophyll a content	0.105	0.002	0.189	0.105	-0.015
Chlorophyll b content	0.110	0.005	0.160	-0.088	0.027
Carotenoida content	0.117	0.064	-0.035	0.064	0.076
Total chlorophyll content	0.112	0.004	0.167	-0.058	0.003
Photosynthetic rate	0.081	-0.093	-0.080	-0.157	0.365
Intercellular CO_2_ concentration	-0.087	-0.117	-0.087	-0.167	0.161
Stomatal conductance	0.084	0.120	-0.018	0.226	-0.211
Transpiration rate	0.085	-0.108	0.028	0.253	-0.171
Superoxide dismutase activity	-0.033	0.166	-0.042	-0.183	-0.153
Peroxidase activity	-0.021	0.146	0.071	0.116	0.446
Catalase activity	-0.025	0.105	0.224	-0.069	0.390
Ascorbate peroxidase	-0.070	0.129	-0.072	0.137	-0.093
Malondialdehyde	-0.043	-0.142	0.131	0.215	-0.206
Hydrogen peroxide	-0.105	0.066	0.011	0.249	0.168
reactive oxygen species	-0.066	0.095	0.044	-0.303	-0.269
Proline	0.041	0.052	-0.238	0.303	0.196
Soluble protein	0.074	0.149	-0.067	-0.013	-0.170
Eigenvalue	7.354	5.086	2.977	1.649	1.162
Contribution (%)	36.771	25.431	14.885	8.244	5.812
Cumulative contribution rate (%)	36.771	62.202	77.087	85.331	91.143

Weights for the five principal components under different flooding stress treatments were calculated using a standard formula, and composite scores (D-values) were derived to rank treatment groups (**Table 3**). The weights for the principal components were 0.403, 0.279, 0.163, 0.090, and 0.064, respectively. Among the four treatment groups, G1-FP ranked highest with a composite score of 0.581, followed by G1-FS (0.533), G2-FP (0.500), and G2-FS (0.354). Overall, flooding tolerance ranked as *Elytrigia elongata* > *Cynodon dactylon × C. transvaalensis ‘Tifdwarf’* under flooding stress alone, while after exogenous spermidine (Spd) application, the ranking reversed to *Elytrigia elongata* > *Cynodon dactylon × C. transvaalensis ‘Tifdwarf’*. These results indicate that *E. elongata* was more tolerant to flooding under both the same inundation conditions and the inundation conditions of externally applied Spd. The external application of Spd under flooding stress effectively improves flooding resistance and mitigates stress injury in both species, with the strongest promotive effect observed in *E. elongata*, followed by *‘Tifdwarf’* (**Table 3**).

**Table 3:** Composite index values, weights, affiliation function values, D-values and flood tolerance rankings of the two ecological grasses.

Groups		Principal component 1		Principal component 2		Principal component 3		Principal component 4		Principal component 5	
G1-FS		-2.165		3.796		0.618		-1.272		0.372	
G1-FP		2.100		1.266		-2.672		1.577		-1.912	
G2-FS		-2.229		-2.590		2.378		0.598		-0.030	
G2-FP		2.294		-2.471		-0.324		-0.903		1.570	

Groups	X(1)	X(2)	X(3)	X(4)	X(5)	D-value	Rank
G1-FS	0.921	2.693	1.725	0.935	1.737	0.533	2
G1-FP	2.232	1.769	0.774	1.689	1.078	0.581	1
G2-FS	0.902	0.360	2.234	1.430	1.621	0.354	4
G2-FP	2.291	0.403	1.453	1.033	2.082	0.500	3
weight	0.403	0.279	0.163	0.090	0.064		

G1. *Elytrigia elongata*, G2. *Cynodon dactylon × C. transvaalensis* ‘Tifdwarf’, FS—waterlogging, FP—waterlogging+Spd;

## 4 Discussion

### 4.1 Effects of different treatments on the main growth indexes of two ecological grasses

Flooding adversely affects soil structure and gas diffusion rates, which can impair root respiration and subsequently inhibit plant growth (Dat et al., 2004). Spd plays a crucial role in plant physiological regulation and stress responses (Pan and Xue, 2012), promoting root elongation and growth by enhancing root cell division (Wei and Newton, 2005). In the present study, FS significantly reduced the shoot fresh weight, root fresh weight, and root length of both *E. elongata* and *‘Tifdwarf’*. These reductions are likely attributable to the anoxic conditions caused by submergence, which lower the oxygen concentration around roots, restrict root respiration, and lead to the accumulation of phytotoxic substances in the soil that inhibit root development (Xin et al., 2012). However, the belowground fresh weight of *E. elongata* continued to increase with prolonged flooding, likely due to its inherent tolerance to waterlogging (Chen et al., 2020), Alternatively, under waterlogging stress, the plant’s root respiration may be impeded, leading to severe energy deficiency. This causes the plant to sacrifice vertical root elongation in favor of promoting the formation of lateral roots or adventitious roots (Sauter, 2013). Moreover, the exogenous application of Spd under FS significantly improved shoot fresh weight, root fresh weight, and root length in both species. These findings suggest that Spd can alleviate the detrimental effects of flooding and promote biomass accumulation, possibly through its known role in delaying senescence and maintaining cellular activity under stress conditions (Li et al., 2017).

### 4.2 The effects of different treatments on the chlorophyll content two ecological grasses

Flooding induces anaerobic respiration, which disrupts chlorophyll biosynthesis and reduces overall chlorophyll content in plants (Ding et al., 2020). Carotenoids play a key role in capturing and transferring light energy to chlorophyll a, serving as important accessory pigments in photosynthesis (Wang et al., 2021). Previous studies have shown that Spd can alleviate blockages in the conversion of chlorophyll precursors, such as from protochlorophyllide to uroporphyrinogen III, thereby promoting chlorophyll accumulation (Xiang et al., 2020), Furthermore, foliar application of Spd has been reported to regulate chlorophyll biosynthesis and degradation pathways, protect against oxidative stress, and improve chlorophyll stability (Sawariya et al., 2024). In this study, we found that FS stress significantly reduced the chlorophyll and carotenoid contents in both ecological grasses, aligning with physiological results reported by Yang et al. (2022). on arborvitae willow seedlings under submergence. In contrast, foliar application of Spd under FS increased chlorophyll a, chlorophyll b, total chlorophyll, and carotenoid contents, likely due to Spd’s capacity to alleviate hypoxia induced degradation of photosynthetic pigments and maintain pigment stability.

### 4.3 The effects of different treatments on the photosynthetic gas exchange parameters of two ecological grasses

Photosynthesis is the primary driver of plant growth and biomass accumulation, providing the energy required for metabolic processes (Flexas and Carriquí, 2020). Under flooding stress, stomatal closure occurs, leading to reduced gas exchange and a consequent decline in photosynthetic rate (Nie et al., 2021). Spd has been shown to enhance photosynthetic capacity under stress conditions, thereby improving plant tolerance to flooding (Cui et al., 2022). In this study, exogenous Spd application under FS conditions reversed these trends suggesting that Spd helps maintain stomatal aperture, enhances photosynthetic activity, and contributes to flood tolerance. These findings are consistent with previous studies, such as that of Hu et al. (2021). which demonstrated that Spd improved the physiological performance of Phoebe bournei seedlings under drought stress through enhanced photosynthetic activity.

### 4.4 Effects of different treatments on the antioxidant response and oxidative stress of two ecological grasses

Flooding stress triggers the accumulation of reactive oxygen species (ROS), leading to potential oxidative damage in plants (Jiang et al., 2013). In response, *E. elongata* activated a comprehensive enzymatic defense system, showing significantly increased activities of superoxide dismutase (SOD), peroxidase (POD), catalase (CAT), and ascorbate peroxidase (APX) under FS, which aligns with findings in spinach and melon under similar conditions (Seymen, 2021; Diao et al., 2020). This coordinated upregulation helped maintain a dynamic ROS balance, contributing to its flooding tolerance (Sun et al., 2018). In contrast, *‘Tifdwarf’* exhibited a divergent response, with increased SOD and CAT activities but decreased POD and APX, suggesting a compensatory mechanism where ROS scavenging primarily relied on SOD and CAT (Tang et al., 2008; Liu et al., 2006).The application of exogenous Spd, FP treatment, played a decisive role in reshaping the oxidative stress response. It significantly reduced the activities of SOD, POD, CAT, and APX in both species. This downregulation was directly linked to the concurrent reduction in measured ROS levels—specifically H_2_O_2_ and superoxide anions—under FP treatment. This evidence supports the mechanism that Spd directly alleviates oxidative stress by neutralizing ROS, potentially through cation chelation or by acting as an H^+^ carrier and participating in disproportionation reactions that partially substitute for SOD (Jin et al., 2010; Wang, 2019). Consequently, the reduced ROS load diminished the demand for high enzymatic antioxidant activity.

The mitigation of oxidative stress by Spd was further confirmed by a reduction in membrane lipid peroxidation. Malondialdehyde (MDA) content, a key indicator of membrane lipid peroxidation and is widely used to assess the extent of oxidative stress and the resilience of plant tissues (Zhao et al., 2023). increased significantly in *‘Tifdwarf’* under FS but was markedly reduced by Spd, consistent with reports by Shi et al. (2016). *E. elongata* displayed a biphasic MDA response under FS—decreasing initially but increasing with prolonged stress—indicating a gradual loss of protection over time (Liang et al., 2019). However, Spd application progressively counteracted this trend, ultimately lowering MDA levels, corroborating the findings of Seng et al. (2012b). The FS induced rise in H_2_O_2_ and superoxide anions, consistent with observations in rice (Ma et al., 2015), was effectively reversed by Spd, aligning with studies showing Spd’s role in suppressing anaerobic respiration and ROS accumulation (Jian et al., 2022).

### 4.5 Effects of different treatments on the contents of free proline and soluble protein in two ecological grasses

Free proline and soluble proteins are key osmoregulatory substances in plants, which accumulate substantially under stress to maintain cellular osmotic balance and confer protection against adverse conditions (Liu et al., 2009). The increase in proline content following flooding is often used as an indicator of plant flooding tolerance, with the timing of peak accumulation reflecting the degree of stress adaptation (Luo et al., 2007). Flooding depth also differentially influences soluble protein content. For instance, Liang et al. (2015) reported that short term shallow flooding promoted soluble protein accumulation in Phragmites australis seedlings, whereas prolonged deep flooding caused a decline. In this study, flooding stress in the present study increased proline content in both ecological grasses, aligning with results from Li et al. (2013) who examined the response of three wetland species to flooding and drought in Dongting Lake, and Song et al. (2013), who investigated flooding effects on root morphology and leaf physiology in soybean cultivars with varying flood tolerance. The soluble protein content in *E. elongata* initially increased and subsequently declined with extended flooding duration, whereas in *‘Tifdwarf’*, soluble protein content decreased continuously, mirroring the pattern reported by Liang et al. (2015) Moreover, exogenous application of Spd enhanced the levels of proline and soluble proteins in both species, suggesting that Spd effectively regulates cellular osmoregulatory substances, stabilizes cell membranes, and maintains osmotic potential under flooding stress. These findings are consistent with those of Wang et al. (2016).

## 5 Conclusions

In summary, the application of 1 mmol/L exogenous Spd effectively mitigated the adverse effects of flooding stress in two ecological grass species. Under flooding conditions, Spd treatment promoted epidermal cell proliferation and biomass accumulation, enhanced photosynthetic capacity by increasing chlorophyll content and gas exchange parameters, and modulated antioxidant enzyme activity. This led to elevated levels of free-state Spd, which scavenged accumulated reactive oxygen species (ROS), while increasing osmoregulatory substances that mitigated oxidative damage to membrane lipids and helped maintain cellular osmotic balance. Consequently, Spd alleviated the inhibitory effects of flooding on the growth of two ecological grasses. Overall, this study demonstrated that flooding stress significantly impairs the growth and physiological functions of the two ecological grasses, whereas exogenous Spd application mitigates these detrimental impacts. These findings provide a theoretical basis for developing strategies to enhance flooding tolerance in grass species and support the potential use of Spd as a practical agent to improve stress resilience in ecological grasslands subjected to waterlogging.

## Data Availability

The original contributions presented in the study are included in the article/supplementary material. Further inquiries can be directed to the corresponding author.
